# Lower airway obstruction due to a massive clot resulting from late bleeding following mini-tracheostomy tube insertion and subsequent clot removal and re-intubation

**DOI:** 10.1186/s40981-017-0087-4

**Published:** 2017-04-17

**Authors:** Hiroshi Inoue, Jun Ito, Hiroaki Uchida, Mariko Morita, Takahiko Masuda, Kazuhiro Yamaya, Masaki Hata, Shigeaki Kato

**Affiliations:** 1 Department of Anesthesia and Critical Care Medicine, Sendai Kousei Hospital, 4-15 Hirosemachi, Aoba-ku, Sendai City, Miyagi 9800873 Japan; 2 Department of Anesthesia, Minamisoma Municipal General Hospital, 2-54-6 Haramachi-ku Takamimachi, Minami-Souma City, Fukushima 9750033 Japan; 3 Department of Cardiovascular Surgery, Sendai Kousei Hospital, 4-15 Hirosemachi, Aoba-ku, Sendai City, Miyagi 9800873 Japan; 4 Cardiovascular Center, Sendai Kousei Hospital, 4-15 Hirosemachi, Aoba-ku, Sendai City, Miyagi 9800873 Japan

**Keywords:** Mini-tracheostomy, Endotracheal tube obstruction, Clot, Lower airway obstruction, Fiberoptic bronchoscopy, Extubation

## Abstract

**Background:**

Easier to perform than the conventional procedure, mini-tracheostomy (MT) is widely used in the operating room or intensive care unit to remove sputum or other obstructions of the upper airway. This option, however, does carry the risk of various complications, including malposition, disposition, bleeding, and subcutaneous emphysema. Here, we report a case of endotracheal tube obstruction due to a massive clot resulting from late bleeding around the insertion site of an MT tube. This necessitated removal of the endotracheal tube together with the clot followed tube re-introduction.

**Case presentation:**

The patient was an 85-year-old man in whom an MT tube had been inserted 6 days earlier following aortic replacement surgery. On re-admittance to our intensive care unit, large amounts of hemosputum and clotting were observed around the insertion site of the tube. The MT tube was subsequently removed and tracheal intubation performed. Ventilation via the endotracheal tube proved impossible, however, and cardiac arrest ensued. Fiberoptic bronchoscopy revealed that the endotracheal tube was completely obstructed by a massive clot. Therefore, we immediately pushed the clot toward the right main bronchus to secure ventilation via the left lung. After many attempts to remove the massive clot, including suction and grasping with basket forceps, it was successfully dislodged by replacing the endotracheal tube with a new one while maintaining oxygenation by one-lung ventilation. Any small fragments of the clot that still remained were then removed by suction under fiberoptic bronchoscopy.

**Conclusions:**

Here, we report a case of endotracheal tube obstruction due to a clot derived from very late (6 days) bleeding after insertion of an MT tube. The patient was successfully rescued by replacing the clot-bearing endotracheal tube with a new one. This experience suggests that the intensive care physician should be aware of the potential risk of clot retention in endotracheal tubes after the elapse of several days.

## Background

Mini-tracheostomy (MT) was first reported in 1984 and is applied in cases of sputum retention [1] or acute airway obstruction [2]. It is usually performed in an operating room or intensive care unit (ICU). Although considered less invasive than an open tracheostomy, an MT carries the risk of complications, including malposition, disposition, bleeding, subcutaneous emphysema, and delayed healing [3–5]. Meanwhile, airway trouble is a major cause of death in the ICU [6]. Here, we report a case of endotracheal tube (ETT) obstruction due to a clot at the insertion site of an MT tube due to very late bleeding.

## Case presentation

**Fig. 1:**
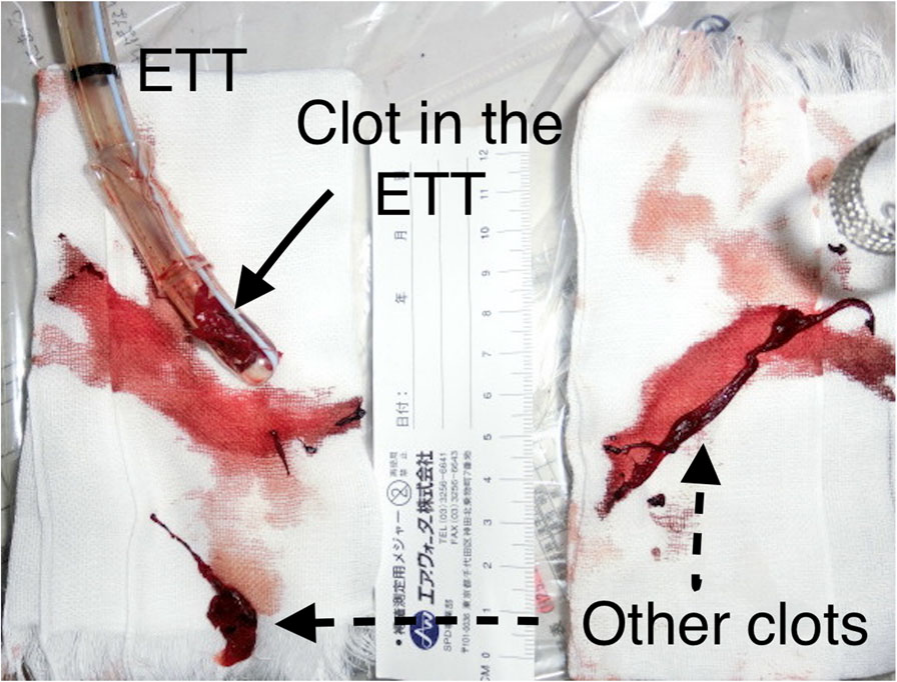
Removed clot and endotracheal tube. The removed clot lodged within the endotracheal tube (*black arrow*); the remaining clot (*dotted arrow*) removed by suction under fiberoptic bronchoscopy. *ETT* endotracheal tube

An 85-year-old man who had undergone ascending aortic replacement developed large amounts of sputum due to postoperative bronchopneumonia. At 16 days postoperatively, an MT with a Portex Mini-Trach® 2 Kit (Smith Medical International: Ashford, Kent, U.K.) was uneventfully performed to alleviate sputum retention, although he was still on warfarin for new onset of sustained arterial fibrillation and arterial flutter. At that time, he was placed on a prothrombin time-international normalization ratio of between 2 and 3. At 22 days, the amount of sputum increased and was, for the first time, accompanied by hemosputum. Fiberoptic bronchoscopy (FOB) revealed a large clot due to bleeding around the insertion site of the MT tube, but there was no bleeding from the bronchial tree. His respiratory condition gradually deteriorated, and his oxygen saturation decreased. After removing the MT tube to avoid interference, tracheal intubation with the ETT (internal diameter of 8.0 mm) was performed. However, immediately after this procedure, the patient’s respiratory condition deteriorated catastrophically and his lung could not be ventilated with a self-expandable respiratory bag. Cardiac arrest soon followed, and cardiopulmonary resuscitation was performed. Direct laryngoscopy reconfirmed that the ETT was securely positioned in the trachea. An FOB examination performed during resuscitation revealed a massive blood clot completely obstructing the ETT. The FOB was used to push the clot away from the ETT toward the right main bronchus. Thereafter, his left lung was successfully ventilated and spontaneous circulation resumed. Any attempts to remove the clot by drawing it out through the ETT consequently failed because it was too big or too solid. Fortunately, oxygenation was preserved via one-lung ventilation during these procedures. Finally, it was decided to replace the ETT after removing the original with the clot still just inside. The clot was subsequently secured with the FOB and basket forceps and pulled into the ETT, after which, both were removed and a new ETT successfully inserted (Fig. 1). Thereafter, other smaller clots and blood were removed with the FOB and absence of bleeding from the bronchial tree confirmed. Open tracheostomy was performed 14 days later. The patient was weaned from mechanical ventilation at 48 days after this event and discharged in satisfactory condition with no psychiatric or neurological damage.

### Discussion

**Fig. 2:**
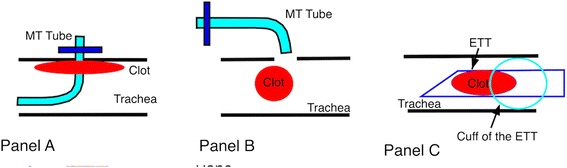
Schematic of the mechanism of occlusion of the endotracheal tube. Suspected three-stage mechanism of airway obstruction. **a** Pre-intubation condition of the airway containing a clot around the insertion site of a mini-tracheostomy tube. **b** The clot falling into the trachea when the mini-tracheostomy tube was removed. **c** Lower airway obstruction caused by a clot. *MT* mini-tracheostomy, *ETT* endotracheal tube

Mini-tracheostomy is performed widely to remove accumulated sputum [1] and acute obstructions of the airway [2]. There is no clear evidence, however, suggesting the superiority of MT over other sputum clearance methods such as medical or physiotherapies. In a review of the management of sputum retention, Beach et al. noted that an MT might be useful along with physiotherapy in adults following thoracic surgery. Its utility in other situations, including an acute care setting, remains unclear [7]. Although an MT is considered less invasive and more easily performable than conventional tracheostomy, it carries the risk of a number of complications [3–5]. Catastrophic airway obstruction due to clotting has been reported, but in most cases, this has occurred during or immediately after insertion of an MT tube [4, 5]. Late bleeding after MT tube insertion can therefore be considered a rare occurrence: indeed, in one case, the latest bleeding time was reported as occurring at approximately 38 h after insertion of an MT tube [8]. In the present case, bleeding appeared to occur at 6 days after insertion, and meanwhile, the patient had not complained of hemosputum. Fiberoptic bronchoscopy revealed a massive clot in the tracheal wall around the insertion site of the MT tube, but not in the lower bronchi (Fig. 2). It was assumed that this clot, which became detached upon tracheal intubation, was obstructing ventilation in the trachea. The etiology of this late bleeding at the insertion site in the present case is unknown at this stage, but we cannot exclude the possibility that it was due to the anti-coagulant effect of the warfarin. Mini-tracheostomy tube insertion is not contraindicated in anticoagulated patients in Japan. However, the present case suggests that MT tube insertion carries the risk of abnormal bleeding at the insertion site in such patients. This indicates the need to be aware of the potential of such an event when performing MT tube insertion in patients receiving anticoagulants.

A number of methods are used to remove clots from the lower airway, including suction, forceps under FOB, a rigid bronchoscope, an arterial embolectomy catheter, and fibrinolytic drugs to the airway.

Bodenham reported the development of a unique suction technique, which involved combining the ETT with a Y-connecter as a suction catheter [9]. If unsuccessful, a number of other options are available. These include rigid bronchoscopy, an arterial embolectomy catheter (i.e., number 6 Fogarty catheter inflated with 1.5 ml of air) [10], and topical thrombolysis. This latter option involves the application of 3000 to 12,000 units of streptokinase [11, 12] or 2500 units of urokinase [13] mixed with normal saline under visualization of the clot via FOB. Replacement of a clot-bearing ETT with a new one offers another option but can only be considered if the condition of the patient allows. Only patients in whom respiratory and/or circulatory conditions are not compromised may be eligible for ETT tube replacement. These techniques are summarized in Table 1.

**Table 1:** Various previously described methods of removing airway clots

Tracheal suction	
Suction with flexible FOB	
Basket forceps under FOB	
Rigid bronchoscope with suction and/or forceps	
Arterial embolectomy passing catheter as far as the distal aspect of the clot	
Topical fibrinolytic drugs administered to the airway	
Removal of the endotracheal tube and clot	

*FOB* fiberoptic bronchoscope

## Conclusions

Here, we reported a rare case of ETT obstruction due to very late bleeding caused by the insertion of an MT tube. Lower airway obstruction is a potentially fatal complication, and the intensive care physician doctor must be aware of the risk of airway troubles due to clot retention in the tubes.

## Abbreviations

ETT: Endotracheal tube
FOB: Fiberoptic bronchoscopy
ICU: Intensive care unit
MT: Mini-tracheostomy
